# Association between postpartum depression and breastfeeding self-efficacy in mothers: a systematic review and meta-analysis

**DOI:** 10.1186/s12884-024-06465-4

**Published:** 2024-04-12

**Authors:** Golnaz Sadat Ahmadinezhad, Fatemeh Zahra Karimi, Mahboobeh Abdollahi, Elham NaviPour

**Affiliations:** 1 School of Nursing and Midwifery, Mashhad university of medical sciences, Mashhad, Iran; 2 Nursing and Midwifery Care Research Center, Mashhad University of Medical Sciences, Mashhad, Iran; 3 Department of Public Health, Torbat Heydarieh University of Medical Sciences, Torbat Heydarieh, Iran; 4 Department of Social Medicine, Faculty of Medicine, Sabzevar University of Medical Sciences, Sabzevar, Iran; 5 Department of Medical Education, Faculty of Medicine, Shiraz University of Medical Sciences, Shiraz, Iran

**Keywords:** Self -efficacy, Breastfeeding, Breastfeeding self -efficacy, Depression, Postpartum depression

## Abstract

**Background:**

Depression is one of the most common mental disorders in the postpartum period. Depression can decrease self-efficacy in breastfeeding by reducing the mother’s self-confidence. Considering the conflicting results regarding the relationship between postpartum depression and breastfeeding self-efficacy, this systematic review was conducted to investigate the relationship between breastfeeding self-efficacy and postpartum depression.

**Method:**

In this systematic review, published articles in PubMed, Scopus, Web of Sciences, Cochrane Library, and Google Scholar databases were searched using English keywords “Self-efficacy, breastfeeding, breastfeeding Self-efficacy, depression, postpartum depression” without publication date limit. Data analysis was done with employing STATA14 software. Heterogeneity was assessed using I^2^ index which was 0%. Therefore, the fixed effects method was used to combine the data and perform meta-analysis.

**Result:**

The results of the meta-analysis showed that based on the fixed effect method, depression was associated with decreased breastfeeding self-efficacy on the first day (SMD = 0.62, 95%CI: -0.830, -0.41, *p* = 0.0001) and on the third day (SMD = 0.84, 95% CI: -0.55,1.14, *p* = 0.0001). The Begg and Manzumdar test revealed no publication bias, with *p* = 0.317.

**Conclusion:**

Postpartum depression may be associated with a decrease in the mother’s breastfeeding self-efficacy and placing mother in a condition to pay low attention to her maternal role. Therefore, healthcare providers should provide adequate support according to the needs of mothers and develop diagnostic and treatment protocols to improve the level of maternal health.

## Background

Breastfeeding guarantees survival, health and growth in all children and improves human capital development [1]. The World Health Organization and the United Nations Children’s Fund have recommended exclusive breastfeeding for all infants from birth to 6 months of age and have emphasized the support and continuation of breastfeeding with the addition of nutritional supplements until the child is two years old [2]. Despite many obvious benefits of breastfeeding for children and the numerous activities of public health centers to promote breastfeeding, the rate of exclusive breastfeeding is low [3]. According to UNICEF, the global average of exclusive breastfeeding in infants under 6 months is 41% [4]. Studies show that in the world, 56% of infants are exclusively breastfed up to 4 months, and 28% of infants are breastfed up to 6 months of age [5, 6]. In Iran, despite planning and effective steps to promote breastfeeding, the rate of exclusive breastfeeding until 6 months of age was estimated 45% in 2016 [6].

Many factors affect mother’s success in breastfeeding and exclusive breastfeeding including mother’s knowledge and attitude, support systems, mother’s social and economic status, and mother’s self-efficacy in breastfeeding [7]. Self-efficacy is the personal belief of people on the extent of their influence on important events in their lives [8]. Self-efficacy is one of the important psychological factors that may affect the duration of breastfeeding [9]. Self-efficacy in Breastfeeding is defined by the mother’s confidence and belief in her ability to breastfeed [10]. Studies show that the main reason for the breastfeeding failure of mothers and the reduction of their self-efficacy in the developing countries is the delay in the initiation of breastfeeding and in the developed countries the shortness of breastfeeding duration [5, 11].

Several factors are effective on mothers’ self-efficacy in breastfeeding such as the mother’s education, type of delivery, family and social support, satisfaction with postpartum care, mother’s understanding of breastfeeding, and mother’s anxiety about breastfeeding [12]. Self-efficacy in breastfeeding is not only influenced by the individual’s performance but also by the mother’s mental health. Therefore, psychological disorders such as stress, anxiety, depression, and reduction of social support can adversely affect the self-efficacy of breastfeeding in mothers [13].

Mothers face an increased risk of mood vulnerability and depression due to changes in such hormones as estrogen, progesterone, prolactin, cortisol, and oxytocin during the postpartum period. Postpartum depression starts 2–4 weeks after delivery and may last for months. Postpartum depression is characterized by such symptoms as crying, sense of helplessness, guilt, insufficient concentration, fatigue, loss of appetite, difficulty in concentration, sleep disorders, and the feeling of inability to deal with the baby [14].

Postpartum depression is associated with significant consequences in mothers, such as remaining weight after delivery, weakness of the functional status, and reducing the quality of communication with the spouse. It can also lead to poor interaction between mother and child which, in turn, may harm the child’s growth and cognitive development [15]. Postpartum depression can affect the mother’s self-confidence, leading to mood depression, decreased interest in activities, difficulty in taking care of the child, feelings of guilt, and difficulty in concentrating, which ultimately leads to a decreased self-efficacy of mothers in breastfeeding [16].

The decrease in self-efficacy of the mother in breastfeeding leads to the failure of the mother in breastfeeding which can cause irreparable complications such as a decrease in mother-baby attachment, and an increase in the incidence of baby’s allergic diseases such as asthma, respiratory infections, obesity, type 1 diabetes, Crohn’s disease and Intestinal infections. Therefore, the continuation of the mother’s breastfeeding will increase the physical and psychological health of the infants [17].

So, postpartum depression, by affecting the self-efficacy of mothers in breastfeeding, can lead to the failure of mothers in breastfeeding and can have many related side effects. Although several studies have examined the relationship between depression and breastfeeding self-efficacy in mothers, there are contradictory studies on the relationship between depression and breastfeeding self-efficacy. In their study, Ngo et al. [18] showed that breastfeeding rate, duration of breastfeeding, mother’s mental health status, and depression are effective on breastfeeding self-efficacy in women. Therefore, increasing the mother’s self-efficacy in breastfeeding can be associated with increasing the mother’s sense of self-satisfaction and well-being, improving mother-baby communication, and increasing the health of the baby [18]. Mercan et al. [19] in their study showed a direct relationship between postpartum depression and breastfeeding self-efficacy. So the more severe the symptoms of postpartum depression, the more self-efficacy of mothers in breastfeeding decreases which endangers the health of the mother and the baby and disrupts the continuation of breastfeeding [19]. While Dias et al. [20] in their study did not report a statistically significant relationship between postpartum depression and the mother’s success in breastfeeding. This can be due to the difference in the social and cultural conditions of the mothers, the choice of type of complementary feeding, and the social and economic conditions governing the society [20].

In addition, to the best knowledge of the researcher, no systematic and comprehensive study has been conducted in this field. A systematic review is a comprehensive review of the literature that systematically and transparently identifies, selects, and critically evaluates all related studies, as well as collects and analyzes data from existing studies [21]. A systematic review and meta-analysis is done with the purpose of systematic and principled examination of evidence, quantitative summarization of the results of each study, combining the results of different studies, and providing a general interpretation of the results [22].

Due to the existence of contradictions in the results of various studies, the current review study was conducted to investigate the relationship between postpartum depression and breastfeeding self-efficacy of mothers.

## Method

The present study, which is a type of systematic review and meta-analysis, was conducted based on the guidelines of the preferred cases in the report of systematic reviews and meta-analysis (PRISMA). This study has been registered with the ethics code: IR.MUMS.NURSE.REC.1402.040 in the ethics committee of Mashhad University of Medical Sciences. For this purpose, the articles indexed in Scopus, Cochrane databases, PubMed, Web of Science databases, Google Scholar, SID, and Magiran, which used any of the English keywords of Self-efficacy, breastfeeding, breastfeeding Self-efficacy depression, postpartum depression and by Boolean operators OR or AND were searched without time limit until the time of the research. To access more information, the references of the reviewed articles were also reviewed to access other related articles. Key terms were verified for appropriateness before the actual search.

### Search strategy

The following search string was employed to retrieve articles from PubMed:

- ((“Breast Feeding“[MeSH Terms] AND ((“associate“[All Fields] OR “associated“[All Fields] OR “associates“[All Fields] OR “associating“[All Fields] OR “association“[MeSH Terms] OR “association“[All Fields] OR “associations“[All Fields]) AND (“depression “[All Fields] OR " postpartum “[All Fields] OR " postpartum depression “[All Fields]))) OR ((“maternally“[All Fields] OR “maternities“[All Fields] OR “maternity“[All Fields] OR “mothers“[MeSH Terms] OR “mothers“[All Fields] OR “maternal“[All Fields]) AND (“self -efficacy “[All Fields] OR " breastfeeding self -efficacy “[All Fields]

### Eligibility and exclusion criteria

Abstracts were extracted and reviewed from all articles. Using the inclusion criteria and exclusion criteria, irrelevant topics were excluded and relevant articles were chosen for the study. The inclusion criteria in this study included the articles published in reliable scientific research journals, with the design type of epidemiological studies consisting of observational and descriptive studies that were either in Farsi or English, and had the keywords of the present study in their title and keywords section. The exclusions criteria included review studies, letters to the editor, articles presented in conferences, case reports, interventional studies, re-reporting of information in the form of a new article, articles with incomplete and unrelated data from the study, and the articles to the to the full text of which there was a lack of access.

Independently, all the articles were searched with the keywords mentioned in the title, abstract, and keywords. After removing duplicate studies, the remaining studies were screened and irrelevant articles were removed. Then, the full texts of the remaining studies were retrieved and the eligible articles were identified and included in the study.

### Study quality

The selected articles were qualitatively evaluated. In each step, if there was any disagreement, the authors would make a final decision by consensus through a discussion with the third researcher. The quality of the articles to be included in the systematic review was assessed by using the Newcastle-Ottawa Quality Assessment Scale for observational studies. To reduce the bias, the quality of the articles was checked by two independent evaluators, and if there was any disagreement between the two evaluators, the article was discussed and reviewed in the presence of another observer to reach a consensus. The Newcastle-Ottawa quality review scale (observational studies version) is a standard scale for evaluating the quality of observational articles. This scale evaluates the articles in terms of the selection (in 4 sections including Representativeness of the sample, Sample size, Non-respondents, and Ascertainment of the exposure), comparability, and outcome (in two sections including Assessment of the outcome and the Statistical test). I If the items considered in the scale were mentioned, it would be scored 1, and if it was not mentioned, it would be scored 0. The total scores assigned to the reported items were considered the article’s total quality score. According to the Newcastle-Ottawa scale, the highest score that each article can get is 10 (the strongest study) and the lowest score is zero (the weakest study). To evaluate the quality, the articles that got a score lower than the average score (less than 4 points) were considered as low quality. Two authors independently collected the required data for the studies and recorded it in the checklist designed by the research team.

### Outcome measures

Extracted data from the articles included: the first author’s name, year of publication, study location, study design, sample size, questionnaire, results, and total score obtained from the Newcastle-Ottawa scale. After collecting the data, the extracted data was reviewed. If there was a different opinion between two researchers about the data, the problem would be referred to the senior researcher, and the final decision would be announced by her. In the first stage, the process of qualitative synthesis of the extracted data was done for systematic review. Then, to perform quantitative data synthesis, the data extracted from the articles that were capable of meta-analysis were entered into Stata14 software.

### Statistical analysis

Considering that the investigated index is the relationship between depression and breastfeeding self-efficacy in mothers, in order to combine the results of different studies, the mean and standard deviation and the standardized mean difference index were used in each study. Heterogeneity was not verified by Chi-squared based on the Q-test and I^2^ statistics. The I^2^ index was 0%; therefore, homogeneity was confirmed and the fixed effects method was used. Sensitivity analysis was used to check the robustness of the meta-analysis results, and Bagg’s test was used to check the publication bias. In case of publication bias, the Trim and Liff method was used to combine studies. To analyze the combination of the results of the studies, the command for analysis was “metan” which was performed using the Stata14 software. *P*-values < 0.05 were taken to be significantly different.

## Results

### Results of literature search and study characteristics

**Fig. 1:**
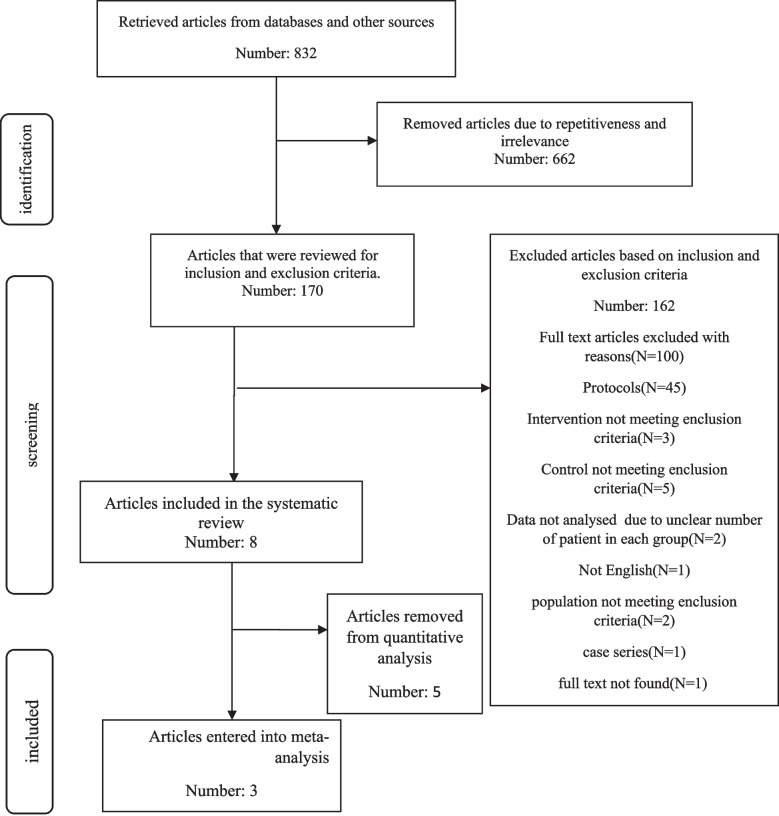
Shows the PRISMA flow chart, which summarizes the literature search, screening, and the number of included studies

In the search of the databases, 864 articles were first retrieved, after removing 704 duplicate and unrelated articles, 160 articles were evaluated based on the inclusion criteria. Finally, after removing 152 cases, 8 full-text articles were included in the systematic review. Afterwards, 5 articles were excluded from the quantitative stage due to incomplete data or no control group, and finally, 3 articles were included in the meta-analysis. The PRISMA flowchart of the review of the studies is shown in Fig. 1.

The characteristics of the studies included in the systematic review and meta-analysis are shown in Table 1. Study design for All studies were cross-sectional and descriptive analyses. The publication year of the studies varied from 2013 to 2021. All of the 8 articles, were published in English language (Table 1). The sample size of 8 selected studies included 1839 people.

**Table 1:** Summary of the included studies

Author / year / reference	Study place	Questionnaire	Sample size	Study design	Results	The score obtained from the Newcastle-Ottawa scale
1. Sahin (2019) [16]	Turkey	breastfeeding self-efficacy questionnaire Edinburgh depression questionnaire	357 breastfeeding mothers	Descriptive-analytical	The mean score of breastfeeding self-efficacy in women was 56.41 ± 8.97. Based on the EPDS total score, mothers who scored 12 or more. were classified as a risk group. 72 mothers (20.2%) are at higher risk of depression. Considering the increased risk of depression, it was found that women with depression have a lower breastfeeding self-efficacy score.	7
2. Zubaran et al(2013) [23]	Australia	breastfeeding self-efficacy questionnaire Edinburgh depression questionnaire	89 breastfeeding mothers	Cross-sectional	For the average breastfeeding self-efficacy, R2 = 0.123 The average score for breastfeeding self-efficacy was 63.51	6
3. Minamida et al(2020) [24].	Japan	breastfeeding self-efficacy questionnaire Edinburgh depression questionnaire	185 breastfeeding mothers	Cross-sectional	Self-efficacy is a protective factor for exclusive breastfeeding, while depression is a risk factor Depression has an inverse relationship with self-efficacy in breastfeeding (*p* < 0.500).	5
4. Mercan and Selcuk (2021) [19]	Turkey	breastfeeding self-efficacy questionnaire Edinburgh depression questionnaire	398 women aged 15–49 in the first 42 days of the postpartum period	A cross-sectional	The average breastfeeding self-efficacy score of mothers was 55.13 ± 8.9. There is a negative statistical relationship between BSES-SF and EPDS scores, so that breastfeeding self-efficacy decreases as postpartum depression increases. β = 0.194 for self-efficacy β = 0.114 for depression	6
5. Aslan and Ege (2016) [25]	Turkey	breastfeeding self-efficacy questionnaire Edinburgh depression questionnaire	265 postpartum women	Descriptive-Analytical	The average self-efficacy score of breastfeeding women is 58.92 ± 7.61 and the average score of the Edinburgh scale is 9.58 ± 5.10. The results of the study showed that one-third of mothers with a low breastfeeding self-efficacy score are at risk of developing depression symptoms.	6
6.Abuchaim ED (2018) [26]	Brazil	breastfeeding self-efficacy questionnaire Edinburgh depression questionnaire	208 women up to 60 days postpartum	Cross-sectional	Postpartum depression symptoms were present in 31.25% of women who presented medium (39.9%) and high (36.06%) levels of breastfeeding self-efficacy.	6
7. Vieira et al (2018) [27]	Brazil	breastfeeding self-efficacy questionnaire Edinburgh depression questionnaire	83 breastfeeding mothers	prospective cohort	self-efficacy is considered a protective factor for exclusive breastfeeding, while depression is a risk factor. Depression has an opposite relationship with self-efficacy in breastfeeding (*p* < 0.500).	5
8. Palancı and Aktaş (2021) [28]	Turkey	breastfeeding self-efficacy questionnaire Edinburgh depression questionnaire	254 mothers with babies aged 2–6 months	Cross-sectional	total mean Breastfeeding self-efficacy scores of mothers were 57.201 ± 7.612 And the average postpartum depression score was 8.516 ± 5.304. A significant relationship was found between breastfeeding self-efficacy and postpartum depression in mothers. R2 = 0.330 for breastfeeding self-efficacy R2=-0.338 for depression	5

**Fig. 2:**
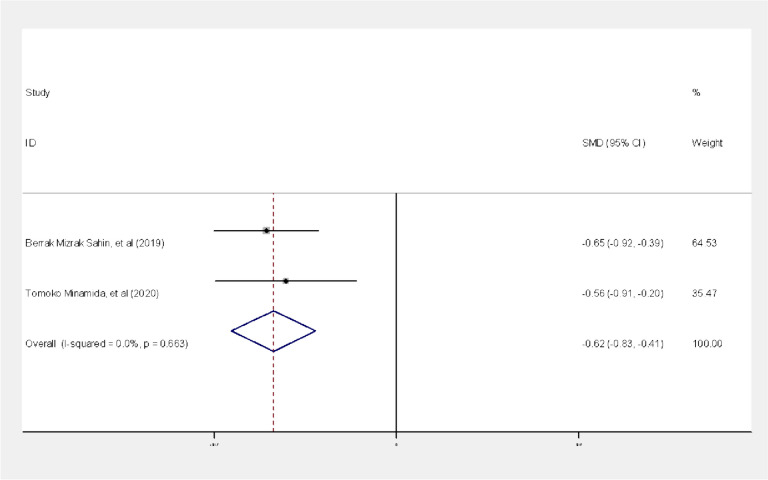
Forest plot of studies

The results of the meta-analysis of the studies of Sahin [16] and Minamida et al. [24] using the standardized mean difference effect size showed that there was homogeneity between the studies based on the I^2^ index (I2 = 0%, *P* = 0.663, Q = 0.19), Therefore, the fixed effect method was used to combine the studies and the final results of the study effects. Based on the results of the fixed effect method, there was a statistically significant difference between the case group (depressed mothers) and the control group (non-depressed mothers) in terms of the mean score of breastfeeding self-efficacy on the first day after delivery (*P* = 0.0001); So that the standardized mean difference between the two groups was estimated as -0.62 with a confidence interval of -0.41, -0.830 (Fig. 2).

In other words, there was a significant difference in the mean score of breastfeeding self-efficacy between the case and control groups, and it was higher in the control group (the group without depression).

Publication bias: In the investigation of this hypothesis, publication bias was not observed as the results of the Begg test revealed the *P*-value = 0.317.

**Fig. 3:**
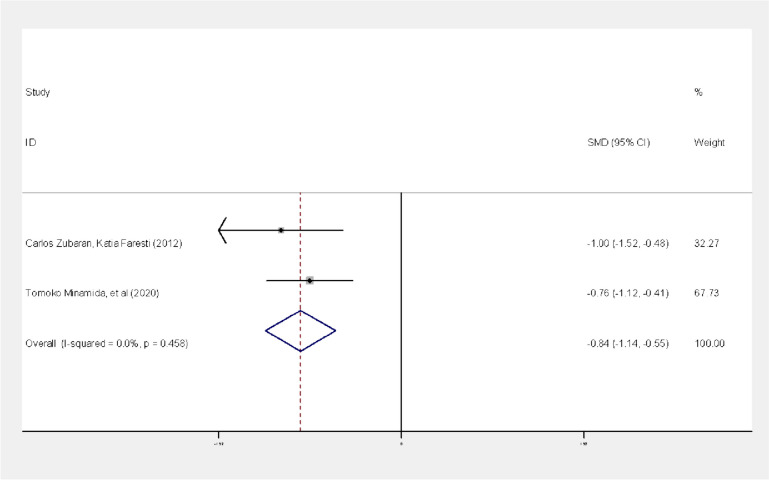
Forest plot of studies

The results of the meta-analysis of the studies of Zubaran and Foresti [23] and Minamida et al. [24] using the standardized mean difference effect size, showed that there was homogeneity between studies based on the I^2^ index (I^2^ = 0%, *P* = 0.458, Q = 0.55). Therefore, the fixed effect method was used to combine the studies and the final results of the study effects. Based on the results of the fixed effect method, there was a statistically significant difference between the two case groups (depressed mothers) and control (non-depressed mothers) in terms of the mean score of breastfeeding self-efficacy on the third day after delivery (*P* = 0.0001); The standardized mean difference between the two groups was estimated as -0.84 with a confidence interval of -0.55, -1.14 (Fig. 3).

In other words, the mean score of breastfeeding self-efficacy in the case and control groups showed a significant difference, and it was higher in the control group (non-depressed mothers).

Publication bias: In the investigation of this hypothesis, publication bias was not observed, as the results of the Begg test reported *P*-value = 0.317.

## Discussion

The results of the present systematic review and meta-analysis showed that postpartum depression can significantly reduce breastfeeding self-efficacy in mothers. Sahin BM [16] also reported that the occurrence of postpartum depression could affect mothers’ ability to take care of the baby and breastfeeding and it reduced the self-efficacy of mothers in breastfeeding [16, 23].

Postpartum depression has a negative effect on the hormones that affect breastfeeding, causing disruption in mothers’ performance and reducing their self-efficacy in breastfeeding [17]. Palancı Ay O et al. [28] also showed a statistically significant relationship between postpartum depression and breastfeeding self-efficacy among mothers in their study, the findings of this study showed that the mother’s age, educational status, number of births, desirability of pregnancy, gender of the baby, history of abortion, and maternal diseases during pregnancy were among the factors that increased the probability of postpartum depression and which could possible lead to a decrease in breastfeeding self-efficacy of mothers [28].

Abuchaim ED et al. [26] showed that there was a negative statistical relationship between breastfeeding self-efficacy and postpartum depression, so that with the increased risk of postpartum depression, the level of breastfeeding self-efficacy of mothers decreased. The first few weeks after birth are considered to be a very important and sensitive period in the formation of the mother-baby relationship. The ability and self-efficacy of the mothers in breastfeeding can lead to the consolidation of the mother-baby relationship and prevent the occurrence of psychological disorders such as postpartum depression [26].

The results of the study by Borra C et al. [29] also indicated that postpartum depression affected the mother’s self-confidence and led to a decrease in her self-efficacy in breastfeeding. In this study, women were examined from the period of pregnancy through 32 weeks after delivery, and the results showed that women who had managed to breastfeed their child were less likely to suffer from postpartum depression [29]. Ngo LTH et al. [18] also showed in their study that the amount of breastfeeding, duration of breastfeeding, mother’s mental health status, and depression were effective on breastfeeding self-efficacy of mothers.

In this study, breastfeeding self-efficacy was related to the mother’s age, occupation, previous experience of breastfeeding, mode of delivery, skin-to-skin contact with the baby, and the amount of breastfeeding in the hospital. Also, the mother’s social support had a significant relationship with postpartum depression. Mercan Y et al. [19] reported that breastfeeding self-efficacy was an important factor in determining the mother’s current intention to breastfeed and her breastfeeding duration.

The results of this study revealed that postpartum depression can have a direct effect on the self-efficacy of mothers in breastfeeding, so that the more severe the symptoms of postpartum depression are, the more the self-efficacy of mothers in breastfeeding decreases [19]. The results of the study by Nishioka et al. [30] in Japan showed that postpartum depression is related to the cessation of breast milk so the cessation of exclusive breastfeeding during the first 5 months of birth causes the appearance of postpartum depression symptoms. However, Dias CC et al. [20] did not report a statistically significant relationship between postpartum depression and the mother’s success in breastfeeding in their study.

In this study, it was known that psychological factors and interpersonal relationships of the mother with those around her were more effective than postpartum depression on the self-efficacy of mothers in breastfeeding, which is not consistent with the results of the present study [20].

The main mechanism of postpartum depression associated with breastfeeding self-efficacy in mothers is not clearly defined. Previous studies show that psychological and social factors such as anxiety, stress, depression, and low self-confidence can have a negative effect on the breastfeeding self-efficacy of mothers [16, 18, 22]. Considering that successful breastfeeding and its continuation is one of the priorities of the World Health Organization and unfavorable performance and feeding with formula can have a negative effect on women’s sense of efficiency. Also, worthiness and self-confidence cause couples to worry about the baby’s health. Moreover, the continuation of the emotional relationship and solidarity between mother and baby and the reduction of related care expenses are among the benefits [23, 24, 28].

Special attention should be paid to diseases and factors affecting breastfeeding and its continuation in mothers. Considering the role of mothers in breastfeeding and its continuity in the baby’s health, the significant prevalence of formula feeding, and the clinical importance of breastfeeding by mothers, it seems necessary to investigate the performance and self-efficacy of mothers in breastfeeding to better understand the dimensions of self-efficacy in mothers and adopt preventive measures. Besides, early education and counseling are necessary for mothers’ breastfeeding behavior [19].

As few studies have so far been conducted on the relationship between postpartum depression and breastfeeding self-efficacy of mothers, the systematic review and meta-analysis of the present study are considered as strengths. In the present study, some articles were excluded from the meta-analysis stage due to the lack of a control group, incomplete reporting of findings, or due to the lack of reporting of the mean and standard deviation of the total breastfeeding self-efficacy score, which was one of the limitations of this study, so the conclusions of the present study should be carefully considered.

## Conclusion

Suffering from postpartum depression can lead to a decrease in breastfeeding self-efficacy of mothers and the continuation of breastfeeding, Also it can cause numerous physical and mental complications for the mother and baby, such as respiratory and digestive infections, necrotizing enterocolitis, insufficient growth and development, and obesity. Continuation of breastfeeding and empowering the mother in breastfeeding and its continuation leads to sufficient growth and development in the baby and has beneficial mental and psychological results such as increasing mother-baby attachment.

Therefore, policymakers and health care providers should identify breastfeeding mothers at risk of depression symptoms and take appropriate interventions such as forming support groups to promote breastfeeding and training family members to support exclusive breastfeeding to encourage mothers to exclusive breastfeeding and reduce depression. It all should be done after delivery to prevent the harmful results of this disorder on the mother, newborn, family and health care system. It is suggested that future studies investigate the factors affecting postpartum depression and breastfeeding self-efficacy of mothers, also intervention studies are suggested to reduce postpartum depression, and counseling and educational intervention studies are recommended to increase mothers’ self-efficacy in breastfeeding.

## Data Availability

The data that support the review findings of current study are available from the corresponding author on reasonable request.
