# Correction: Association between postpartum depression and breastfeeding self-efficacy in mothers: a systematic review and meta-analysis

**DOI:** 10.1186/s12884-024-06576-y

**Published:** 2024-05-17

**Authors:** Golnaz Sadat Ahmadinezhad, Fatemeh Zahra Karimi, Mahboobeh Abdollahi, Elham NaviPour

**Affiliations:** 1 School of Nursing and Midwifery, Mashhad university of medical sciences, Mashhad, Iran; 2 Nursing and Midwifery Care Research Center, Mashhad University of Medical Sciences, Mashhad, Iran; 3 Department of Public Health, Torbat Heydarieh University of Medical Sciences, Torbat Heydarieh, Iran; 4 Department of Social Medicine, Faculty of Medicine, Sabzevar University of Medical Sciences, Sabzevar, Iran; 5 Department of Medical Education, Faculty of Medicine, Shiraz University of Medical Sciences, Shiraz, Iran

**Correction: BMC Pregnancy Childbirth 24, 273 (2024)**

10.1186/s12884-024-06465-4

Following publication of the original article [1], the authors reported an error in the Method part related to the article’s Ethics code.

The incorrect code is IR.MUMS.NURSE.REC.1402.044.

The correct code is IR.MUMS.NURSE.REC.1402.040.

The original article has been corrected.
